# Pseudosymmetric *fac*-di­aqua­trichlorido[(di­methyl­phosphor­yl)methanaminium-κ*O*]manganese(II)

**DOI:** 10.1107/S1600536813008751

**Published:** 2013-04-10

**Authors:** Guido J. Reiss

**Affiliations:** a Institut für Anorganische Chemie und Strukturchemie, Lehrstuhl II: Material- und Strukturforschung, Heinrich-Heine-Universität Düsseldorf, Universitätsstrasse 1, D-40225 Düsseldorf, Germany

## Abstract

In the title compound, [Mn(C_3_H_11_NOP)Cl_3_(H_2_O)_2_], the Mn^II^ metal center has a distorted o­cta­hedral geometry, coordinated by the three chloride ligands showing a facial arrangement. Two water mol­ecules and the *O*-coordinated dpmaH cation [dpmaH = (di­methyl­phosphor­yl)methanaminium] complete the coordination sphere. Each complex mol­ecule is connected to its neighbours by O—H⋯Cl and N—H⋯Cl hydrogen bonds. Two of the chloride ligands and the two water ligands form a hydrogen-bonded polymeric sheet in the *ab* plane. Furthermore, these planes are connected to adjacent planes by hydrogen bonds from the aminium function of cationic dpmaH ligand. A pseudo-mirror plane perpendicular to the *b* axis in the chiral space group *P*2_1_ is observed together with inversion twinning [ratio = 0.864 (5):0.136 (5)].

## Related literature

For related dpma compounds, see: Borisov *et al.* (1994 ▶); Kochel (2009 ▶); Reiss & Jörgens (2012 ▶). For a definition of the term tecton, see: Brunet *et al.* (1997 ▶). For the use of anionic phosphinic acid derivatives as supra­molecular tectons, see: Glidewell *et al.* (2000 ▶); Chen *et al.* (2010 ▶). For related methyl­phosphinic acids and derivatives, see: Reiss & Engel (2008 ▶); Meyer *et al.* (2010 ▶). For graph-set theory and its applications, see: Etter *et al.* (1990 ▶); Bernstein *et al.* (1995 ▶); Grell *et al.* (2002 ▶). For related manganese complexes, see: Głowiak & Sawka-Dobrowolska (1977 ▶); Feist *et al.* (1997 ▶); Kubíček *et al.* (2003 ▶); Karthikeyan *et al.* (2011 ▶). For manganese complexes as model system for metalloproteins, see: Wieghardt (1989 ▶). For examples of pseudo-symmetry, see: Jones *et al.* (1988 ▶); Reiss (2002*a* ▶,*b* ▶); Reiss & Konietzny (2002 ▶); Ruck (2000 ▶).
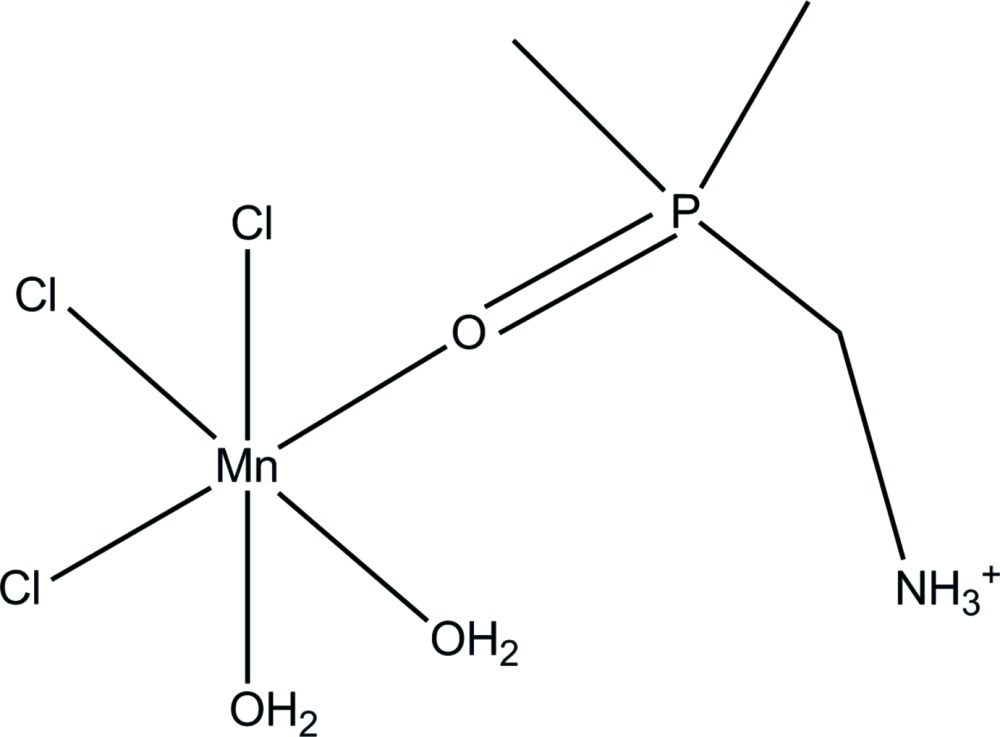


## Experimental

### 

#### Crystal data

[Mn(C_3_H_11_NOP)Cl_3_(H_2_O)_2_]
*M* *_r_* = 305.42
Monoclinic, 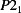

*a* = 6.3535 (3) Å
*b* = 10.7304 (6) Å
*c* = 8.5629 (4) Å
β = 99.490 (2)°
*V* = 575.79 (5) Å^3^
*Z* = 2
Mo *K*α radiation
μ = 1.95 mm^−1^
*T* = 290 K
0.41 × 0.30 × 0.26 mm

#### Data collection

Bruker APEXII CCD diffractometer
Absorption correction: multi-scan (*SADABS*; Bruker, 2008 ▶) *T*_min_ = 0.723, *T*_max_ = 0.980
29900 measured reflections
4538 independent reflections
4518 reflections with *I* > 2σ(*I*)
*R*_int_ = 0.030

#### Refinement

*R*[*F* ^2^ > 2σ(*F* ^2^)] = 0.014
*wR*(*F* ^2^) = 0.038
*S* = 1.11
4538 reflections
149 parameters
5 restraints
H atoms treated by a mixture of independent and constrained refinement
Δρ_max_ = 0.49 e Å^−3^
Δρ_min_ = −0.29 e Å^−3^
Absolute structure: Flack (1983 ▶), 2165 Friedel pairs
Flack parameter: 0.136 (5)

### 

Data collection: *APEX2* (Bruker, 2008 ▶); cell refinement: *SAINT* (Bruker, 2008 ▶); data reduction: *SAINT*; program(s) used to solve structure: *SHELXS97* (Sheldrick, 2008 ▶); program(s) used to refine structure: *SHELXL97* (Sheldrick, 2008 ▶); molecular graphics: *DIAMOND* (Brandenburg, 2011) ▶; software used to prepare material for publication: *publCIF* (Westrip, 2010 ▶).

## Supplementary Material

Crystal structure: contains datablock(s) I, global. DOI: 10.1107/S1600536813008751/gg2113sup1.cif

Structure factors: contains datablock(s) I. DOI: 10.1107/S1600536813008751/gg2113Isup2.hkl

Supplementary material file. DOI: 10.1107/S1600536813008751/gg2113Isup3.mol

Additional supplementary materials:  crystallographic information; 3D view; checkCIF report

## Figures and Tables

**Table 1:** Hydrogen-bond geometry (Å, °)

*D*—H⋯*A*	*D*—H	H⋯*A*	*D*⋯*A*	*D*—H⋯*A*
N1—H11⋯Cl2^i^	0.928 (19)	2.402 (19)	3.3220 (10)	171.3 (15)
N1—H12⋯Cl2^ii^	0.82 (2)	2.56 (2)	3.2664 (9)	145.9 (19)
N1—H13⋯Cl3^ii^	0.899 (17)	2.436 (17)	3.2193 (8)	145.8 (14)
O1*W*—H1*O*⋯Cl3^iii^	0.84 (1)	2.42 (1)	3.2360 (8)	164 (2)
O1*W*—H2*O*⋯Cl1^iv^	0.86 (1)	2.37 (1)	3.2021 (7)	164 (2)
O2*W*—H3*O*⋯Cl1^iii^	0.82 (1)	2.39 (1)	3.2026 (8)	171 (2)
O2*W*—H4*O*⋯Cl3^ii^	0.84 (1)	2.35 (1)	3.1635 (8)	166 (2)

Symmetry codes: (i) 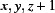; (ii) 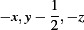; (iii) 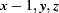; (iv) 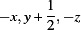.

**Fig. 1.**
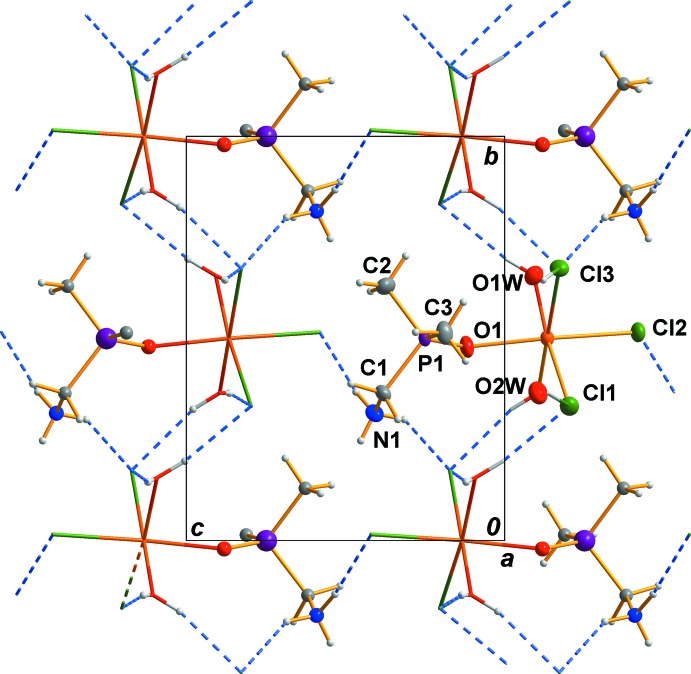
View against the a axis on the hydrogen bonding scheme of the title structure (I). The atoms of the asymmetric unit is labelled and drawn as 45% ellipsoids.

**Fig. 2.**
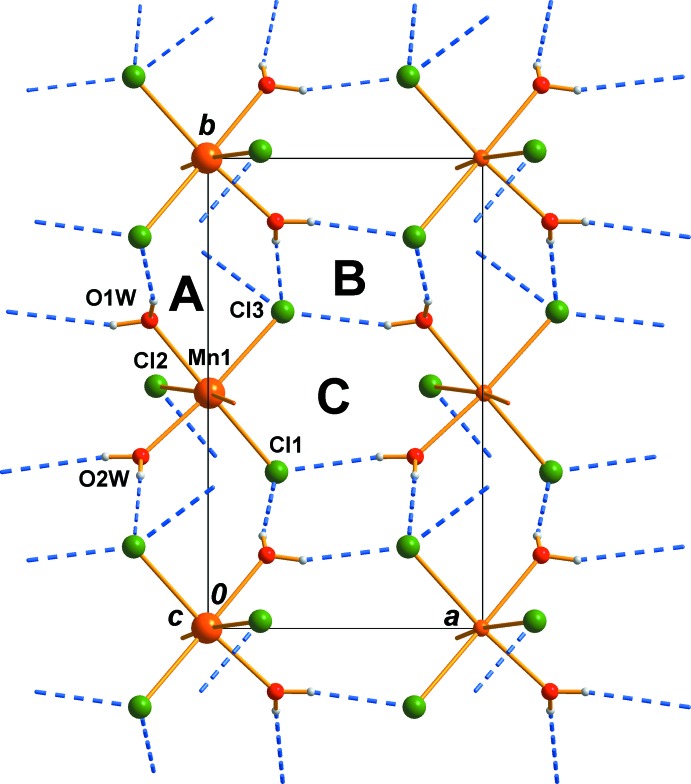
View along [001] on a two-dimensional segment of the title structure.Three different hydrogen bonded ring motifs (A, B, C) are shown which are all belonging to the R22(8) graph-set type.

**Fig. 3.**
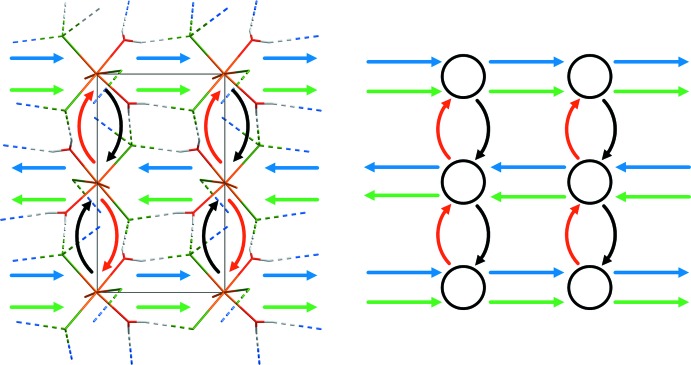
Left part: Wireframe Model of the title structure with the hydrogen bonds shown as arrows. Right part: Constructor-graph (Grell et al., 2002) for the same part of the title structure (large black dots represent the Mn complexes; colour-codes arrows indicate the crystallographically independent hydrogen bonds).

**Fig. 4.**
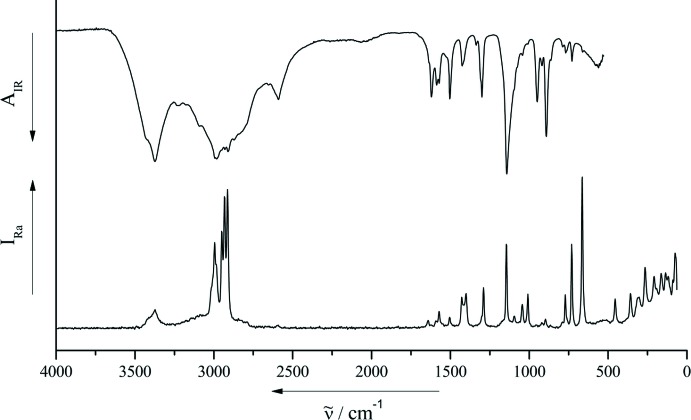
Showing the Raman- and the infrared spectra of the title compound.

## supplementary crystallographic information

### Comment

Manganese complexes are of general interest as these metal centers play important roles in biological systems such as metalloproteins (Wieghardt, 1989). More than one hundred manganese complexes built by aqua, chlorido and any organic donor ligands at the same time are structurally characterized and deposited in the Cambridge Structural Database. If we limit the search on compounds with at least one aqua, one chloride and a ligand with a *O*-coordinated phosphoryl group the number is reduced to only two examples (Głowiak & Sawka-Dobrowolska, 1977; Kubíček *et al.*, 2003) which are comparable with the title complex. Furthermore, alkyldiphosphinates are known to be efficient tectons (for the term tecton, see: Brunet *et al.*, 1997) to construct hydrogen bonded frameworks (Glidewell *et al.*, 2000). Especially it has also been shown that amino phosphinic anions are able to form hydrogen bonded one-dimensional, two-dimensional and three-dimensional supramolecular architectures (Chen *et al.*, 2010). For the neutral *dmpa* there are some examples that show its ability to coordinate transition metals (Borisov *et al.* 1994; Kochel, 2009) and also a salt containing the protonated *dpma*H cation has been structurally characterized (Reiss & Jörgens, 2012). The structure determination on *fac*-diaquatrichlorido((dimethylphosphoryl)methanaminium)manganese(II) is part of our continuing interest in the hydrogen bonding of methylphosphinic acids and its derivatives (Reiss & Engel, 2008) and the field of application as a tectons for the construction of new hydrogen bonded networks (*e.g.* Meyer *et al.*, 2010).

The title structure crystallizes in the monoclinic, chiral space group *P*2_1_. The asymmetric unit consists of one formula unit of the *fac*-diaquatrichlorido((dimethylphosphoryl)methanaminium)manganese(II) complex. The three chlorido ligands show a facial arrangement with Mn—Cl distances between 2.5137 (3) and 2.5717 (3) Å which is in excellent agreement with other Mn(II) complexes (Głowiak & Sawka-Dobrowolska, 1977; Kubíček *et al.*, 2003; Karthikeyan *et al.*, 2011). The Cl—Mn—Cl angles of 92.163 (8)° to 93.346 (8)° are in the typical range of hexacoordinate aqua-chlorido manganese(II) complexes (*e.g.* Feist *et al.* 1997). The distorted octahedral coordination at the Mn(II) metal center is completed by two water molecules and a *O*-coordinated *dpmaH* cation. All Mn—O bond length as well as the geometrical parameters of the *dpma*H cation are in the expected ranges. Each manganese complex is connected to adjacent complexes by O–H···Cl and N—H···Cl hydrogen bonds. Two of the chlorido ligands and the two water ligands form a hydrogen bonded two-dimensional polymer in the *ab* plane (Fig. 1). Adjacent layers are connected to each other by the cationic *dpma*H ligand which is located between them. In detail the *dpma*H ligand coordinates the manganese of one layer by its oxygen atom and forms a hydrogen bond to the next layer by its aminium group. The hydrogen bonding scheme of the formal two-dimensional polymer in the *ab* plane is characterized by three different types of annealed rings (Fig. 2; A, B and C-ring). All rings are classified to belong to the *R*^2^_2_(8) graph-set descriptor (Etter *et al.*, 1990, Bernstein *et al.*, 1995), but they are different in detail. Ring A and B show a pseudo-inversion symmetry (for a more general introduction into pseudo-symmetry, see: Ruck, 2000) whereas ring C seems to have a mirror symmetry. The pseudo-symmetry features of the hydrogen bonding motifs are related to a pseudo-mirror plane present perpendicular to the *b* axis. According to the checkcif algorithm more than 90% of the atom positions of the title structure fulfill this additional symmetry element. Figure 1 visualizes this pseudosymmetry situation and it is abundantly clear that the aminium group significantly breaks this additional symmetry element. Especially in pseudosymmetric cases where no additional (super-structure) reflections are present a close look on the plausibility of structural model (Reiss, 2002*a*; Reiss, 2002*b*; Reiss & Konietzny, 2002;) and on the difference density maps are needed (Jones *et al.*, 1988). In the latter stages of the refinement the presence of inversion twinning (ratio: 0.864 (5) / 0.136 (5)) was detected. The general hydrogen bonding scheme within the *ab* plane can be abstracted by a so-called constructor graph (Fig. 3; Grell *et al.*, 2002). Especially in the constructor graph of the title structure the pseudosymmetry can be clearly seen. The infrared and Raman spectra of the title compound are shown in Fig. 4. Both spectra show bands similar to those reported for *dpma*HCl (Reiss & Jörgens, 2012). A further assignment, especially for the far-infrared region of the Raman spectrum, is difficult as several lines are observed which may belong to modes of the *dpma* ligand or may result from stretch modes of Mn–O and Mn–Cl bonds.

### Experimental

For the synthesis of the title compound (I) equimolar amounts of *dpma* and manganese(II)chloride tetrahydrate were dissolved in concentrated HCl. Slow evaporation of this solution at room temperature yielded crystals suitable for a crystallographic structure determination.

### Refinement

Methyl H-atoms were identified in difference syntheses, idealized and refined using rigid groups allowed to rotate about the P—C bond (AFIX 137 option of the *SHELXL97* program). The coordinates of all other H-atoms were refined freely with individual *U*_iso_values.

### Crystal data

**Table:**

[Mn(C_3_H_11_NOP)Cl_3_(H_2_O)_2_]	*F*(000) = 310
*M**_r_* = 305.42	*D*_x_ = 1.762 Mg m^−^^3^
Monoclinic, *P*2_1_	Mo *K*α radiation, λ = 0.71073 Å
Hall symbol: P 2yb	Cell parameters from 9869 reflections
*a* = 6.3535 (3) Å	θ = 3.1–33.6°
*b* = 10.7304 (6) Å	µ = 1.95 mm^−^^1^
*c* = 8.5629 (4) Å	*T* = 290 K
β = 99.490 (2)°	Block, colourless
*V* = 575.79 (5) Å^3^	0.41 × 0.30 × 0.26 mm
*Z* = 2	

### Data collection

**Table:**

Bruker APEXII CCD diffractometer	4538 independent reflections
Radiation source: fine-focus sealed tube	4518 reflections with *I* > 2σ(*I*)
Graphite monochromator	*R*_int_ = 0.030
φ and ω scans	θ_max_ = 33.6°, θ_min_ = 2.4°
Absorption correction: multi-scan (*SADABS*; Bruker, 2008)	*h* = −9→9
*T*_min_ = 0.723, *T*_max_ = 0.980	*k* = −16→16
29900 measured reflections	*l* = −13→13

### Refinement

**Table:**

Refinement on *F*^2^	Hydrogen site location: inferred from neighbouring sites
Least-squares matrix: full	H atoms treated by a mixture of independent and constrained refinement
*R*[*F*^2^ > 2σ(*F*^2^)] = 0.014	*w* = 1/[σ^2^(*F*_o_^2^) + (0.0222*P*)^2^ + 0.0303*P*] where *P* = (*F*_o_^2^ + 2*F*_c_^2^)/3
*wR*(*F*^2^) = 0.038	(Δ/σ)_max_ = 0.001
*S* = 1.11	Δρ_max_ = 0.49 e Å^−^^3^
4538 reflections	Δρ_min_ = −0.29 e Å^−^^3^
149 parameters	Extinction correction: *SHELXL97* (Sheldrick, 2008), Fc^*^=kFc[1+0.001xFc^2^λ^3^/sin(2θ)]^-1/4^
5 restraints	Extinction coefficient: 0.273 (3)
Primary atom site location: structure-invariant direct methods	Absolute structure: Flack (1983), 2165 Friedel pairs
Secondary atom site location: difference Fourier map	Flack parameter: 0.136 (5)

### Special details

**Table:**

Geometry. All e.s.d.'s (except the e.s.d. in the dihedral angle between two l.s. planes) are estimated using the full covariance matrix. The cell e.s.d.'s are taken into account individually in the estimation of e.s.d.'s in distances, angles and torsion angles; correlations between e.s.d.'s in cell parameters are only used when they are defined by crystal symmetry. An approximate (isotropic) treatment of cell e.s.d.'s is used for estimating e.s.d.'s involving l.s. planes.
Refinement. Refinement of *F*^2^ against ALL reflections. The weighted *R*-factor *wR* and goodness of fit *S* are based on *F*^2^, conventional *R*-factors *R* are based on *F*, with *F* set to zero for negative *F*^2^. The threshold expression of *F*^2^ > σ(*F*^2^) is used only for calculating *R*-factors(gt) *etc*. and is not relevant to the choice of reflections for refinement. *R*-factors based on *F*^2^ are statistically about twice as large as those based on *F*, and *R*- factors based on ALL data will be even larger.

### Fractional atomic coordinates and isotropic or equivalent isotropic displacement parameters (Å^2^)

**Table:**

	*x*	*y*	*z*	*U*_iso_*/*U*_eq_	
Mn1	0.004766 (16)	0.500568 (11)	−0.133762 (11)	0.01871 (3)	
Cl1	0.24906 (3)	0.33264 (2)	−0.20350 (3)	0.02689 (4)	
Cl2	−0.18984 (3)	0.51538 (2)	−0.41916 (2)	0.02810 (5)	
Cl3	0.27145 (3)	0.673730 (19)	−0.17200 (3)	0.02618 (4)	
P1	0.27696 (3)	0.49973 (2)	0.251182 (18)	0.01833 (4)	
O1	0.09298 (10)	0.47944 (7)	0.11869 (7)	0.02894 (13)	
N1	0.09847 (14)	0.31507 (8)	0.40879 (10)	0.02992 (15)	
H11	0.025 (3)	0.3676 (17)	0.467 (2)	0.047 (5)*	
H12	0.114 (3)	0.2472 (18)	0.452 (3)	0.054 (5)*	
H13	0.017 (3)	0.2997 (16)	0.315 (2)	0.039 (4)*	
O1W	−0.21359 (12)	0.65566 (7)	−0.09214 (9)	0.02909 (14)	
O2W	−0.24546 (13)	0.36541 (8)	−0.10488 (9)	0.03152 (14)	
H1O	−0.3455 (15)	0.645 (2)	−0.121 (2)	0.057 (6)*	
H2O	−0.199 (3)	0.6966 (14)	−0.0049 (13)	0.039 (4)*	
H3O	−0.3747 (15)	0.365 (2)	−0.135 (2)	0.058 (6)*	
H4O	−0.247 (4)	0.325 (2)	−0.0213 (19)	0.079 (7)*	
C1	0.30694 (15)	0.36461 (8)	0.38191 (10)	0.02448 (15)	
H11A	0.3837	0.2998	0.3358	0.040 (4)*	
H12A	0.3909	0.3879	0.4828	0.027 (3)*	
C2	0.2410 (2)	0.62945 (10)	0.37378 (13)	0.0359 (2)	
H21	0.2240	0.7039	0.3108	0.100 (9)*	
H22	0.3636	0.6378	0.4552	0.096 (9)*	
H23	0.1161	0.6166	0.4214	0.099 (9)*	
C3	0.52844 (13)	0.51400 (13)	0.18863 (10)	0.03147 (18)	
H31	0.5500	0.4443	0.1227	0.090 (9)*	
H32	0.6393	0.5155	0.2796	0.080 (6)*	
H33	0.5322	0.5899	0.1297	0.058 (6)*	

### Atomic displacement parameters (Å^2^)

**Table:**

	*U*^11^	*U*^22^	*U*^33^	*U*^12^	*U*^13^	*U*^23^
Mn1	0.01908 (5)	0.02042 (5)	0.01630 (5)	−0.00011 (4)	0.00192 (3)	0.00049 (4)
Cl1	0.02524 (9)	0.02786 (9)	0.02755 (8)	0.00643 (7)	0.00433 (6)	0.00031 (7)
Cl2	0.02977 (8)	0.03377 (11)	0.01848 (7)	−0.00040 (7)	−0.00279 (6)	−0.00007 (6)
Cl3	0.02148 (8)	0.02487 (9)	0.03136 (9)	−0.00303 (6)	0.00192 (6)	0.00126 (7)
P1	0.02142 (7)	0.02078 (7)	0.01289 (6)	−0.00034 (7)	0.00309 (5)	0.00136 (7)
O1	0.0264 (3)	0.0411 (4)	0.0174 (2)	−0.0039 (2)	−0.00188 (19)	0.0042 (2)
N1	0.0326 (4)	0.0270 (3)	0.0289 (3)	−0.0090 (3)	0.0016 (3)	0.0044 (3)
O1W	0.0248 (3)	0.0324 (3)	0.0302 (3)	0.0055 (2)	0.0050 (2)	−0.0052 (3)
O2W	0.0267 (3)	0.0393 (4)	0.0281 (3)	−0.0097 (3)	0.0033 (2)	0.0044 (3)
C1	0.0276 (4)	0.0229 (3)	0.0220 (3)	−0.0003 (3)	0.0015 (3)	0.0050 (2)
C2	0.0516 (6)	0.0257 (4)	0.0328 (4)	0.0026 (4)	0.0135 (4)	−0.0048 (3)
C3	0.0238 (3)	0.0482 (6)	0.0234 (3)	−0.0028 (4)	0.0067 (2)	0.0035 (4)

### Geometric parameters (Å, º)

**Table:**

Mn1—O1	2.1538 (6)	N1—H13	0.899 (17)
Mn1—O2W	2.1959 (8)	O1W—H1O	0.841 (9)
Mn1—O1W	2.2326 (7)	O1W—H2O	0.858 (9)
Mn1—Cl1	2.5137 (3)	O2W—H3O	0.820 (9)
Mn1—Cl2	2.5554 (2)	O2W—H4O	0.836 (9)
Mn1—Cl3	2.5717 (3)	C1—H11A	0.9700
P1—O1	1.5046 (6)	C1—H12A	0.9700
P1—C3	1.7735 (8)	C2—H21	0.9600
P1—C2	1.7806 (10)	C2—H22	0.9600
P1—C1	1.8225 (8)	C2—H23	0.9600
N1—C1	1.4800 (12)	C3—H31	0.9600
N1—H11	0.928 (19)	C3—H32	0.9600
N1—H12	0.82 (2)	C3—H33	0.9600
			
O1—Mn1—O2W	83.73 (3)	H11—N1—H13	109.2 (15)
O1—Mn1—O1W	89.08 (3)	H12—N1—H13	104.7 (18)
O2W—Mn1—O1W	89.65 (3)	Mn1—O1W—H1O	118.0 (15)
O1—Mn1—Cl1	95.41 (2)	Mn1—O1W—H2O	122.5 (11)
O2W—Mn1—Cl1	92.30 (2)	H1O—O1W—H2O	106.6 (17)
O1W—Mn1—Cl1	175.27 (2)	Mn1—O2W—H3O	132.3 (15)
O1—Mn1—Cl2	166.216 (19)	Mn1—O2W—H4O	123.2 (17)
O2W—Mn1—Cl2	84.47 (2)	H3O—O2W—H4O	97 (2)
O1W—Mn1—Cl2	83.74 (2)	N1—C1—P1	112.07 (6)
Cl1—Mn1—Cl2	92.163 (8)	N1—C1—H11A	109.2
O1—Mn1—Cl3	97.814 (19)	P1—C1—H11A	109.2
O2W—Mn1—Cl3	174.88 (2)	N1—C1—H12A	109.2
O1W—Mn1—Cl3	85.50 (2)	P1—C1—H12A	109.2
Cl1—Mn1—Cl3	92.413 (8)	H11A—C1—H12A	107.9
Cl2—Mn1—Cl3	93.346 (8)	P1—C2—H21	109.5
O1—P1—C3	114.29 (4)	P1—C2—H22	109.5
O1—P1—C2	113.48 (5)	H21—C2—H22	109.5
C3—P1—C2	108.67 (6)	P1—C2—H23	109.5
O1—P1—C1	109.73 (4)	H21—C2—H23	109.5
C3—P1—C1	104.24 (5)	H22—C2—H23	109.5
C2—P1—C1	105.69 (4)	P1—C3—H31	109.5
P1—O1—Mn1	141.52 (4)	P1—C3—H32	109.5
C1—N1—H11	113.9 (11)	H31—C3—H32	109.5
C1—N1—H12	110.3 (14)	P1—C3—H33	109.5
H11—N1—H12	109.2 (18)	H31—C3—H33	109.5
C1—N1—H13	109.1 (11)	H32—C3—H33	109.5
			
C3—P1—O1—Mn1	−19.09 (9)	Cl2—Mn1—O1—P1	−168.19 (4)
C2—P1—O1—Mn1	106.29 (8)	Cl3—Mn1—O1—P1	−24.42 (7)
C1—P1—O1—Mn1	−135.75 (7)	O1—P1—C1—N1	−40.28 (7)
O2W—Mn1—O1—P1	160.50 (8)	C3—P1—C1—N1	−163.10 (7)
O1W—Mn1—O1—P1	−109.76 (8)	C2—P1—C1—N1	82.43 (8)
Cl1—Mn1—O1—P1	68.77 (7)		

### Hydrogen-bond geometry (Å, º)

**Table:**

*D*—H···*A*	*D*—H	H···*A*	*D*···*A*	*D*—H···*A*
N1—H11···Cl2^i^	0.928 (19)	2.402 (19)	3.3220 (10)	171.3 (15)
N1—H12···Cl2^ii^	0.82 (2)	2.56 (2)	3.2664 (9)	145.9 (19)
N1—H13···Cl3^ii^	0.899 (17)	2.436 (17)	3.2193 (8)	145.8 (14)
O1*W*—H1*O*···Cl3^iii^	0.84 (1)	2.42 (1)	3.2360 (8)	164 (2)
O1*W*—H2*O*···Cl1^iv^	0.86 (1)	2.37 (1)	3.2021 (7)	164 (2)
O2*W*—H3*O*···Cl1^iii^	0.82 (1)	2.39 (1)	3.2026 (8)	171 (2)
O2*W*—H4*O*···Cl3^ii^	0.84 (1)	2.35 (1)	3.1635 (8)	166 (2)

Symmetry codes: (i) *x*, *y*, *z*+1; (ii) −*x*, *y*−1/2, −*z*; (iii) *x*−1, *y*, *z*; (iv) −*x*, *y*+1/2, −*z*.

## References

[bb1] Bernstein, J., Davis, R. E., Shimoni, L. & Chang, N.-L. (1995). *Angew. Chem. Int. Ed.* **34**, 1555–1573.

[bb2] Borisov, G., Varbanov, S. G., Venanzi, L. M., Albinati, A. & Demartin, F. (1994). *Inorg. Chem.* **33**, 5430–5437.

[bb26] Brandenburg, K. (2011). *DIAMOND* Crystal Impact GbR, Bonn, Germany.

[bb3] Bruker (2008). *SADABS*, *SMART* and *SAINT* Bruker AXS Inc., Madison, Wisconsin, USA.

[bb4] Brunet, P., Simard, M. & Wuest, J. D. (1997). *J. Am. Chem. Soc.* **119**, 2737–2738.

[bb5] Chen, S.-P., Zhang, Y.-Q., Hu, L., He, H.-Z. & Yuan, L.-J. (2010). *CrystEngComm*, **12**, 3327–3336.

[bb6] Etter, M. C., MacDonald, J. C. & Bernstein, J. (1990). *Acta Cryst.* B**46**, 256–262. 10.1107/s0108768189012929 2344397

[bb7] Feist, M., Troyanov, S., Stiewe, A., Kemnitz, E. & Kunze, R. (1997). *Z. Naturforsch. Teil B*, **52**, 1094–1102.

[bb8] Flack, H. D. (1983). *Acta Cryst.* A**39**, 876–881.

[bb9] Glidewell, C., Ferguson, G. & Lough, A. J. (2000). *Acta Cryst.* C**56**, 855–858. 10.1107/s0108270100004650 10935106

[bb10] Głowiak, T. & Sawka-Dobrowolska, W. (1977). *Acta Cryst.* B**33**, 2763–2766.

[bb11] Grell, J., Bernstein, J. & Tinhofer, G. (2002). *Crystallogr. Rev.* **8**, 1–56.

[bb12] Jones, P. G., Schelbach, R., Schwarzmann, E. & Thöne, C. (1988). *Acta Cryst.* C**44**, 1196–1198.

[bb13] Karthikeyan, M., Karthikeyan, S. & Manimaran, B. (2011). *Acta Cryst.* E**67**, m1367. 10.1107/S160053681103546X PMC3201495 22064913

[bb14] Kochel, A. (2009). *Inorg. Chim. Acta*, **362**, 1379–1382.

[bb15] Kubíček, V., Vojtíček, P., Rudovský, J., Hermann, P. & Lukeš, I. (2003). *Dalton Trans.* pp. 3927–3938.

[bb16] Meyer, M. K., Graf, J. & Reiss, G. J. (2010). *Z. Naturforsch. Teil B*, **65**, 1462–1466.

[bb17] Reiss, G. J. (2002*a*). *Z. Kristallogr.* **217**, 550–556.

[bb18] Reiss, G. J. (2002*b*). *Z. Natuforsch. Teil B*, **57**, 479–482.

[bb19] Reiss, G. J. & Engel, J. S. (2008). *Acta Cryst.* E**64**, o400. 10.1107/S1600536807068122 PMC2960156 21201428

[bb20] Reiss, G. J. & Jörgens, S. (2012). *Acta Cryst.* E**68**, o2899–o2900. 10.1107/S1600536812037890 PMC3470250 23125694

[bb21] Reiss, G. J. & Konietzny, S. (2002). *J. Chem. Soc. Dalton Trans.* pp. 862–864.

[bb22] Ruck, M. (2000). *Z. Kristallogr.* **215**, 148–156.

[bb23] Sheldrick, G. M. (2008). *Acta Cryst.* A**64**, 112–122. 10.1107/S0108767307043930 18156677

[bb24] Westrip, S. P. (2010). *J. Appl. Cryst.* **43**, 920–925.

[bb25] Wieghardt, K. (1989). *Angew. Chem. Int. Ed.* **28**, 1153–1172.

